# Crystal structure and theoretical study of *N*,*N*-bis­[(5-chloro-2-oxo-2,3-di­hydro­benzo[*d*]oxazol-3-yl)meth­yl]-2-phenyl­ethanamine

**DOI:** 10.1107/S2056989018005984

**Published:** 2018-04-27

**Authors:** Abdullah Aydın, Zeynep Soyer, Mehmet Akkurt, Orhan Büyükgüngör

**Affiliations:** a Department of Mathematics and Science Education, Faculty of Education, Kastamonu University, 37200 Kastamonu, Turkey; b Department of Pharmaceutical Chemistry, Faculty of Pharmacy, Ege University, 35100 Izmir, Turkey; c Department of Physics, Faculty of Sciences, Erciyes University, 38039 Kayseri, Turkey; d Department of Physics, Faculty of Arts and Sciences, Ondokuz Mayıs University, 55139 Samsun, Turkey

**Keywords:** crystal structure, 2,3-di­hydro-1,3-benzoxazole ring, semi-empirical *CNDO*/2 method, HOMO, LUMO

## Abstract

In the mol­ecular structure of the title compound, C_24_H_19_Cl_2_N_3_O_4_, the three C atoms of the central *N*,*N*-di­methyl­methanamine moiety are bonded to the N atoms of the two 5-chloro-1,3-benzoxazol-2(3*H*)-one groups and to the methyl C atom of the methyl­benzene group. One of the nine-membered 2,3-di­hydro-1,3-benzoxazole rings and the phenyl ring are almost parallel to each other, making a dihedral angle of 5.30 (18)°, but they are almost normal to the mean plane of the other nine-membered 2,3-di­hydro-1,3-benzoxazole ring, subtending dihedral angles of 89.29 (16) and 85.41 (18)°, respectively. The crystal structure features C—H⋯O hydrogen bonds and π–π stacking inter­actions [centroid-to-centroid distances = 3.5788 (19) Å, slippage = 0.438 and 3.7773 (16) Å, and slippage = 0.716 Å].

## Abstract 2

One of the nine-membered 2,3-di­hydro-1,3-benzoxazole rings and the phenyl ring are almost parallel to each other, making a dihedral angle of 5.30 (18)°. These rings are almost normal to the mean plane of the other nine-membered 2,3-di­hydro-1,3-benzoxazole ring. The crystal structure features C—H⋯O hydrogen bonds and π–π stacking inter­actions.

## Chemical context   

2(3*H*)-Benzoxazolone is a privileged lead mol­ecule for the design of potential bioactive agents, and its derivatives have been shown to posses a broad spectrum of bioactive properties such as anti-HIV (Deng *et al.*, 2006 ▸), anti­cancer (Ivanova *et al.*, 2007 ▸), analgesic (Ünlü *et al.*, 2003 ▸), anti-inflammatory (Köksal *et al.*, 2005 ▸), anti­nociceptive (Önkol *et al.*, 2001 ▸), anti­microbial (Köksal *et al.*, 2002 ▸), anti­convulsant (Ucar *et al.*, 1998 ▸), anti­malarial (Courtois *et al.*, 2004 ▸) and human leukocyte MPO clorinating inhibitor activities (Soyer *et al.*, 2005 ▸). In this context, we have investigated another benzoxazolone derivative with formula C_24_H_19_Cl_2_N_3_O_4_, and report here its synthesis, mol­ecular, crystal and theoretical structures.
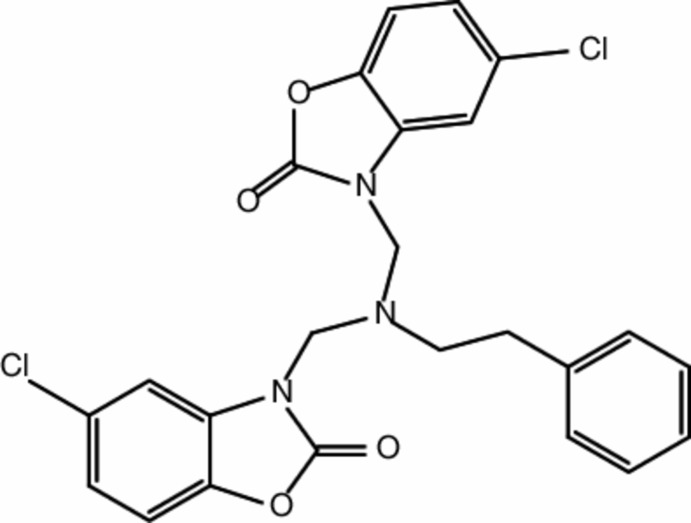


## Structural commentary   

**Figure 1:**
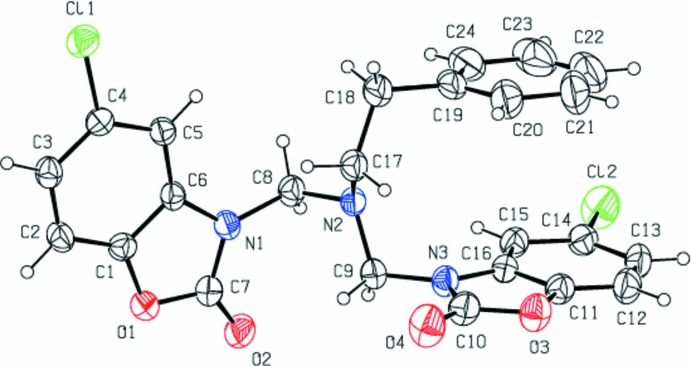
View of the title mol­ecule with the atom-numbering scheme. Displacement ellipsoids for non-H atoms are drawn at the 30% probability level.

The central part of the title mol­ecule (Fig. 1 ▸) comprises an *N*,*N*-di­methyl­methanamine unit whose three carbon atoms are bonded to the N atoms of the two 5-chloro-1,3-benzoxazol-2(3*H*)-one moieties and to the methyl carbon atom of the methyl­benzene group. The nine-membered 2,3-di­hydro-1,3-benzoxazole ring (N3/O3/C10–C16) and the phenyl ring (C19–C24) are almost parallel to each other, making a dihedral angle of 5.30 (18)°. These two entities are almost normal to the mean plane of the other 2,3-di­hydro-1,3-benzoxazole ring (N1/O1/C1–C7), subtending dihedral angles of 89.29 (16) and 85.41 (18)°, respectively.

The C7—N1—C8—N2, N2—C9—N3—C16, N2—C17—C18—C19 and C17—C18—C19—C24 torsion angles are −90.7 (3), −75.6 (3), −63.6 (3) and 106.1 (4)°, respectively. The bond lengths and angles of the title mol­ecule (Table 1 ▸) are normal and correspond to those observed in related benzoxazolone derivatives (Aydın *et al.*, 2004 ▸, 2012 ▸, 2017 ▸; Allen *et al.*, 1987 ▸).

## Supra­molecular features   

**Figure 2:**
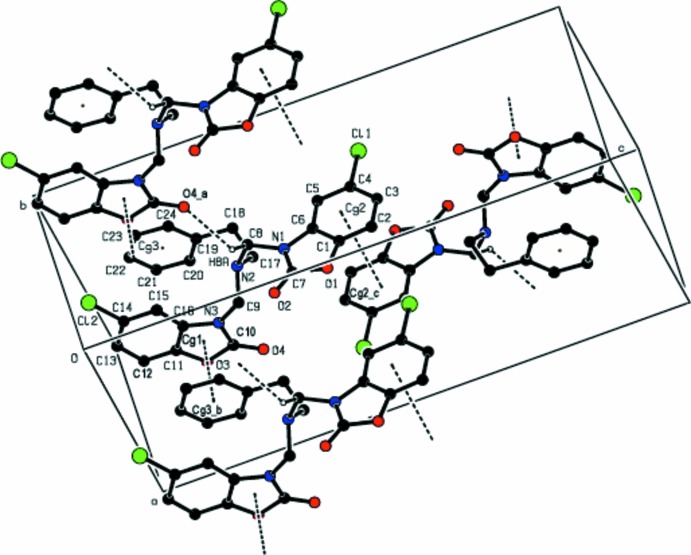
A view of the crystal packing in the title structure, showing the C—H⋯O hydrogen bonding and π–π stacking inter­actions. H atoms not involved in hydrogen bonds are omitted for the sake of clarity. [Symmetry codes: (*a*) *x* − 1, *y*, *z*; (*b*) *x*, *y* − 1, *z*; (*c*) 1 − *x*, 1 − *y*, 1 − *z*.]

The crystal structure features weak inter­molecular C—H⋯O hydrogen bonds (Table 2 ▸, Fig. 2 ▸) between a methyl­ene group and a carbonyl O atom of a neighbouring mol­ecule. π–π stacking inter­actions [*Cg*1⋯*Cg*3^ii^ = 3.5788 (19) Å, slippage = 0.438 Å and *Cg*2⋯*Cg*2^iii^ = 3.7773 (16) Å, slippage = 0.716 Å, symmetry codes: (ii) *x*, −1 + *y*, *z*, (iii) 1 − *x*, 1 − *y*, 1 − *z*, where *Cg*1, *Cg*2 and *Cg*3 are the centroids of the O3/N3/C10/C11/C16 2,3-di­hydro-1,3-oxazole ring, the C1–C6 benzene ring and the C19–C24 phenyl ring, respectively] additionally consolidate the crystal packing.

## Theoretical calculations   

**Figure 3:**
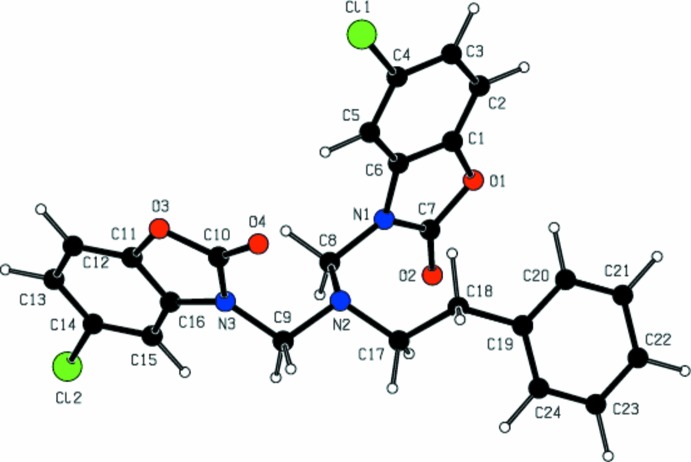
The mol­ecular structure of the title compound calculated using the *CNDO/2* method.

Semi-empirical mol­ecular orbital (MO) calculations of the title mol­ecule were carried out using the *CNDO/2* method (Pople & Segal, 1966 ▸). It is based on the *Complete Neglect of Differential Overlap* integral approximation. The semi-empirical *CNDO/2* parameterization is widely used to derive bond lengths, bond angles, torsion angles, atom charges, *HOMO* and *LUMO* energy levels, dipole moments, polarizability, *etc*. The spatial view of the title compound calculated as a closed-shell in a vacuum at 0 K is shown in Fig. 3 ▸.

In the title mol­ecule, the calculated charges on the Cl1, Cl2, O1, O2, O3, O4, N1, N2 and N3 atoms are −0.164, −0.226, −0.424, −0.228, −0.431, −0.117, −0.187 and −0.112 e^−^, respectively. The calculated dipole moment is about 2.122 Debye. The *HOMO* and *LUMO* energy levels are −10.7480 and 3.4691 eV, respectively.

The calculated bond lengths and angles of the title mol­ecule are consistent with those obtained by X-ray structure determination within error limits (Table 1 ▸). Looking at Figs. 1 ▸ and 3 ▸, the experimental and calculated conformations appear to be quite different. This is supported by the torsion angles N1—C8—N2—C17 [experimental 70.5 (3), calculated 58.25°], N1—C8—N2—C9 [−62.8 (3), −177.78°], N2—C17—C18—C19 [−63.6 (3), −150.35°], C18—C17—N2—C8 [84.1 (3), −95.53°] and C9—N2—C17—18 [143.5 (2), −140.65°]. The small differences between the theoretical and experimental results are due to the calculations being in a vacuum and at 0 K.

## Synthesis and crystallization   

4-Chloro-2-amino­phenol (10 mmol), urea (50 mmol) and 37%_wt_ HCl (2.5 ml) were irradiated (300 W, 413 K) for 15 min in a microwave oven. After completion of the reaction (monitored with TLC), water (10 ml) was added to the reaction mixture and stirred at room temperature for 1 h. The resulting precipitate was filtered and washed with water. After drying the precipitate, crystallization from ethanol–water (1:1 *v*/*v*) yielded 5-chloro-2(3*H*)-benzoxazolone. This compound (2 mmol) was dissolved in methanol (5 ml). Phenethyl­amine (2 mmol) and 37%_wt_ formalin (2.5 mmol) were added to this solution. The mixture was then stirred vigorously for 1 h. The resulting precipitate was filtered and washed with cold methanol. The crude product was recrystallized from methanol, yield 40%; m.p. 427 K.

IR υ_max_ (FTIR/ATR): 3062, 2862, 1769, 1038 cm^−1^; ^1^H NMR (CDCl_3_): δ 2.79 (2H, *t*, *J* = 6.8 Hz, NCH_2_CH_2_), 3.14 (2H, *t*, *J* = 7.0 Hz, CH_2_CH_2_-phen­yl) 4.90 (4H, *s*, 2 × CH_2_), 6.88–7.16 (11H, *m*, Ar-H) ppm; MS (ESI) *m*/*z* (%): 315 (100), 317 (37), 484 (*M* + H, 3), 486 (*M* + H + 2, 1).

## Refinement   

Crystal data, data collection and structure refinement details are summarized in Table 3 ▸. All H atoms were positioned geometrically and allowed to ride on their parent atoms, with C—H = 0.93 (aromatic) and 0.97 (methyl­ene) Å and *U*_iso_ = 1.2*U*_eq_(C).

## Supplementary Material

Crystal structure: contains datablock(s) global, I. DOI: 10.1107/S2056989018005984/wm5440sup1.cif

Structure factors: contains datablock(s) I. DOI: 10.1107/S2056989018005984/wm5440Isup2.hkl

Supporting information file. DOI: 10.1107/S2056989018005984/wm5440Isup3.cml

CCDC reference: 1838126

Additional supporting information:  crystallographic information; 3D view; checkCIF report

## Figures and Tables

**Table 1:** Comparison of experimental (X-ray) and theoretical (*CNDO*/2) bond lenghts and angles (Å, °) for the title compound

Bond	X-ray	*CNDO*/2
Cl1—C4	1.735 (3)	1.7379
Cl2—C14	1.738 (3)	1.7382
O1—C1	1.382 (3)	1.3545
O1—C7	1.384 (3)	1.3585
O2—C7	1.202 (3)	1.2091
O3—C10	1.371 (3)	1.3573
O3—C11	1.390 (4)	1.3544
O4—C10	1.200 (4)	1.2090
N1—C6	1.398 (3)	1.3649
N1—C7	1.371 (3)	1.3593
N1—C8	1.491 (3)	1.4587
N2—C8	1.430 (4)	1.4666
N2—C9	1.448 (4)	1.4641
N2—C17	1.463 (4)	1.4672
N3—C9	1.444 (3)	1.4601
N3—C10	1.370 (4)	1.3587
N3—C16	1.393 (3)	1.3663
C17—C18	1.520 (4)	1.5425
C18—C19	1.506 (4)	1.5131
		
C8—N2—C9	112.5 (2)	110.47
C8—N2—C17	114.76 (19)	112.03
C9—N2—C17	114.5 (2)	110.74
Cl1—C4—C3	118.03 (19)	120.09
Cl1—C4—C5	118.6 (2)	119.73
O1—C7—O2	123.0 (3)	124.49
O2—C7—N1	129.6 (3)	127.02
N1—C8—N2	115.9 (2)	112.31
N2—C9—N3	111.3 (2)	111.17
O3—C10—O4	123.5 (3)	124.70
O4—C10—N3	128.3 (3)	126.73
Cl2—C14—C13	118.4 (2)	120.07
Cl2—C14—C15	118.6 (2)	119.71
N2—C17—C18	111.8 (2)	112.23
C17—C18—C19	112.9 (2)	114.03
C18—C19—C20	121.2 (3)	120.60
C18—C19—C24	121.2 (3)	121.28

**Table 2:** Hydrogen-bond geometry (Å, °)

*D*—H⋯*A*	*D*—H	H⋯*A*	*D*⋯*A*	*D*—H⋯*A*
C8—H8*A*⋯O4^i^	0.97	2.51	3.037 (4)	114

Symmetry code: (i) 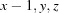.

**Table 3:** Experimental details

Crystal data	
Chemical formula	C_24_H_19_Cl_2_N_3_O_4_
*M*_r_	484.32
Crystal system, space group	Triclinic, *P* 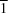
Temperature (K)	296
*a*, *b*, *c* (Å)	7.4028 (5), 7.4432 (5), 22.4616 (15)
α, β, γ (°)	97.255 (5), 90.274 (5), 114.784 (5)
*V* (Å^3^)	1112.36 (14)
*Z*	2
Radiation type	Mo *K*α
μ (mm^−1^)	0.33
Crystal size (mm)	0.61 × 0.26 × 0.04

Data collection	
Diffractometer	Stoe IPDS 2
Absorption correction	Integration (*X-RED32*; Stoe & Cie, 2002 ▸)
*T*_min_, *T*_max_	0.901, 0.987
No. of measured, independent and observed [*I* > 2σ(*I*)] reflections	15409, 4604, 2261
*R*_int_	0.083
(sin θ/λ)_max_ (Å^−1^)	0.628

Refinement	
*R*[*F* ^2^ > 2σ(*F* ^2^)], *wR*(*F* ^2^), *S*	0.043, 0.089, 0.87
No. of reflections	4604
No. of parameters	298
H-atom treatment	H-atom parameters constrained
Δρ_max_, Δρ_min_ (e Å^−3^)	0.13, −0.17

Computer programs: *X-AREA* and *X-RED32* (Stoe & Cie, 2002 ▸), *SHELXS97* and *SHELXL97* (Sheldrick, 2008 ▸), *ORTEP-3 for Windows* and *WinGX* (Farrugia, 2012 ▸) and *PLATON* (Spek, 2009 ▸).

## supplementary crystallographic information

### Crystal data

**Table:**

C_24_H_19_Cl_2_N_3_O_4_	*Z* = 2
*M**_r_* = 484.32	*F*(000) = 500
Triclinic, *P*1	*D*_x_ = 1.446 Mg m^−^^3^
Hall symbol: -P 1	Mo *K*α radiation, λ = 0.71073 Å
*a* = 7.4028 (5) Å	Cell parameters from 12292 reflections
*b* = 7.4432 (5) Å	θ = 1.8–27.2°
*c* = 22.4616 (15) Å	µ = 0.33 mm^−^^1^
α = 97.255 (5)°	*T* = 296 K
β = 90.274 (5)°	Plate, light yellow
γ = 114.784 (5)°	0.61 × 0.26 × 0.04 mm
*V* = 1112.36 (14) Å^3^	

### Data collection

**Table:**

Stoe IPDS 2 diffractometer	4604 independent reflections
Radiation source: sealed X-ray tube, 12 x 0.4 mm long-fine focus	2261 reflections with *I* > 2σ(*I*)
Plane graphite monochromator	*R*_int_ = 0.083
Detector resolution: 6.67 pixels mm^-1^	θ_max_ = 26.5°, θ_min_ = 1.8°
ω scans	*h* = −9→9
Absorption correction: integration (XRED-32; Stoe & Cie, 2002)	*k* = −9→9
*T*_min_ = 0.901, *T*_max_ = 0.987	*l* = −28→28
15409 measured reflections	

### Refinement

**Table:**

Refinement on *F*^2^	Primary atom site location: structure-invariant direct methods
Least-squares matrix: full	Secondary atom site location: difference Fourier map
*R*[*F*^2^ > 2σ(*F*^2^)] = 0.043	Hydrogen site location: inferred from neighbouring sites
*wR*(*F*^2^) = 0.089	H-atom parameters constrained
*S* = 0.87	*w* = 1/[σ^2^(*F*_o_^2^) + (0.0273*P*)^2^] where *P* = (*F*_o_^2^ + 2*F*_c_^2^)/3
4604 reflections	(Δ/σ)_max_ < 0.001
298 parameters	Δρ_max_ = 0.13 e Å^−^^3^
0 restraints	Δρ_min_ = −0.17 e Å^−^^3^

### Special details

**Table:**

Geometry. Bond distances, angles etc. have been calculated using the rounded fractional coordinates. All su's are estimated from the variances of the (full) variance-covariance matrix. The cell esds are taken into account in the estimation of distances, angles and torsion angles
Refinement. Refinement on F^2^ for ALL reflections except those flagged by the user for potential systematic errors. Weighted R-factors wR and all goodnesses of fit S are based on F^2^, conventional R-factors R are based on F, with F set to zero for negative F^2^. The observed criterion of F^2^ > 2sigma(F^2^) is used only for calculating -R-factor-obs etc. and is not relevant to the choice of reflections for refinement. R-factors based on F^2^ are statistically about twice as large as those based on F, and R-factors based on ALL data will be even larger.

### Fractional atomic coordinates and isotropic or equivalent isotropic displacement parameters (Å^2^)

**Table:**

	*x*	*y*	*z*	*U*_iso_*/*U*_eq_	
Cl1	0.33895 (11)	0.91821 (11)	0.55979 (3)	0.0695 (3)	
Cl2	−0.03618 (13)	0.22527 (15)	0.03733 (4)	0.0993 (4)	
O1	0.1417 (3)	0.1051 (3)	0.45148 (8)	0.0614 (7)	
O2	0.0466 (3)	−0.0234 (3)	0.35320 (9)	0.0774 (8)	
O3	0.7070 (3)	0.3740 (3)	0.16410 (8)	0.0729 (8)	
O4	0.7524 (3)	0.3741 (4)	0.26394 (9)	0.0931 (10)	
N1	0.1491 (3)	0.3147 (3)	0.38837 (9)	0.0519 (7)	
N2	0.2740 (3)	0.4370 (3)	0.29248 (8)	0.0487 (8)	
N3	0.4467 (3)	0.3171 (3)	0.21984 (9)	0.0542 (8)	
C1	0.1949 (3)	0.2919 (4)	0.48420 (11)	0.0502 (9)	
C2	0.2325 (4)	0.3444 (4)	0.54484 (11)	0.0567 (10)	
C3	0.2776 (3)	0.5405 (4)	0.56779 (11)	0.0548 (9)	
C4	0.2845 (3)	0.6740 (4)	0.52899 (11)	0.0490 (9)	
C5	0.2442 (3)	0.6204 (4)	0.46734 (11)	0.0487 (9)	
C6	0.2011 (3)	0.4255 (4)	0.44584 (10)	0.0465 (9)	
C7	0.1063 (4)	0.1186 (4)	0.39202 (12)	0.0598 (11)	
C8	0.1117 (4)	0.3782 (4)	0.33094 (10)	0.0548 (9)	
C9	0.3282 (4)	0.2752 (4)	0.27152 (11)	0.0560 (10)	
C10	0.6446 (4)	0.3553 (4)	0.22130 (13)	0.0671 (11)	
C11	0.5420 (4)	0.3407 (4)	0.12702 (12)	0.0570 (10)	
C12	0.5348 (4)	0.3392 (5)	0.06646 (13)	0.0723 (11)	
C13	0.3512 (5)	0.2999 (5)	0.03966 (12)	0.0706 (11)	
C14	0.1891 (4)	0.2687 (4)	0.07364 (12)	0.0622 (11)	
C15	0.1980 (4)	0.2725 (4)	0.13546 (12)	0.0575 (10)	
C16	0.3809 (4)	0.3077 (4)	0.16085 (10)	0.0507 (9)	
C17	0.4427 (4)	0.6267 (4)	0.31468 (11)	0.0577 (10)	
C18	0.4109 (5)	0.8042 (4)	0.29870 (11)	0.0722 (11)	
C19	0.3978 (5)	0.8067 (4)	0.23191 (13)	0.0697 (11)	
C20	0.5647 (6)	0.8579 (5)	0.19940 (15)	0.0932 (14)	
C21	0.5538 (8)	0.8637 (6)	0.13801 (19)	0.122 (2)	
C22	0.3763 (11)	0.8152 (7)	0.1095 (2)	0.136 (3)	
C23	0.2082 (9)	0.7639 (6)	0.1404 (2)	0.121 (2)	
C24	0.2183 (6)	0.7582 (5)	0.20128 (15)	0.0887 (14)	
H2	0.22790	0.25200	0.56970	0.0680*	
H3	0.30310	0.58280	0.60900	0.0660*	
H5	0.24630	0.71120	0.44220	0.0580*	
H8A	−0.00220	0.26870	0.30880	0.0660*	
H8B	0.07580	0.48940	0.34060	0.0660*	
H9A	0.40320	0.25540	0.30360	0.0670*	
H9B	0.20820	0.15270	0.26090	0.0670*	
H12	0.64670	0.36320	0.04440	0.0870*	
H13	0.33700	0.29450	−0.00180	0.0850*	
H15	0.08810	0.25270	0.15810	0.0690*	
H17A	0.56300	0.62780	0.29750	0.0690*	
H17B	0.46100	0.63870	0.35800	0.0690*	
H18A	0.28880	0.80080	0.31520	0.0870*	
H18B	0.52020	0.92670	0.31720	0.0870*	
H20	0.68740	0.88940	0.21890	0.1120*	
H21	0.66850	0.90090	0.11680	0.1460*	
H22	0.36810	0.81670	0.06830	0.1640*	
H23	0.08630	0.73270	0.12030	0.1450*	
H24	0.10250	0.72110	0.22200	0.1070*	

### Atomic displacement parameters (Å^2^)

**Table:**

	*U*^11^	*U*^22^	*U*^33^	*U*^12^	*U*^13^	*U*^23^
Cl1	0.0777 (5)	0.0615 (5)	0.0641 (5)	0.0269 (4)	0.0050 (4)	−0.0006 (4)
Cl2	0.0934 (6)	0.1463 (9)	0.0720 (6)	0.0683 (6)	−0.0183 (5)	0.0007 (5)
O1	0.0774 (12)	0.0546 (12)	0.0547 (12)	0.0289 (10)	0.0104 (9)	0.0128 (10)
O2	0.1047 (16)	0.0566 (13)	0.0598 (13)	0.0252 (12)	0.0167 (11)	0.0014 (11)
O3	0.0563 (11)	0.1035 (16)	0.0651 (13)	0.0394 (12)	0.0080 (10)	0.0133 (12)
O4	0.0797 (15)	0.142 (2)	0.0701 (14)	0.0601 (15)	−0.0131 (12)	0.0120 (14)
N1	0.0590 (13)	0.0511 (13)	0.0451 (12)	0.0215 (11)	0.0062 (10)	0.0117 (10)
N2	0.0526 (13)	0.0542 (14)	0.0412 (12)	0.0238 (12)	0.0059 (10)	0.0094 (10)
N3	0.0562 (14)	0.0671 (15)	0.0434 (13)	0.0297 (12)	0.0032 (10)	0.0090 (11)
C1	0.0504 (15)	0.0532 (17)	0.0506 (16)	0.0255 (14)	0.0034 (12)	0.0068 (13)
C2	0.0639 (17)	0.0652 (19)	0.0477 (16)	0.0313 (15)	0.0018 (13)	0.0170 (14)
C3	0.0542 (15)	0.0677 (19)	0.0441 (14)	0.0268 (15)	0.0000 (12)	0.0100 (14)
C4	0.0458 (15)	0.0503 (16)	0.0505 (16)	0.0204 (13)	0.0030 (12)	0.0051 (13)
C5	0.0485 (14)	0.0529 (16)	0.0472 (15)	0.0224 (13)	0.0068 (11)	0.0127 (12)
C6	0.0449 (14)	0.0559 (17)	0.0414 (15)	0.0226 (13)	0.0077 (11)	0.0119 (13)
C7	0.0707 (19)	0.061 (2)	0.0515 (18)	0.0301 (17)	0.0141 (14)	0.0125 (15)
C8	0.0573 (16)	0.0670 (18)	0.0434 (14)	0.0294 (15)	0.0018 (12)	0.0086 (13)
C9	0.0674 (17)	0.0600 (18)	0.0432 (15)	0.0283 (15)	0.0062 (13)	0.0117 (13)
C10	0.0627 (19)	0.084 (2)	0.0610 (19)	0.0375 (17)	0.0037 (15)	0.0097 (17)
C11	0.0551 (17)	0.0645 (18)	0.0553 (17)	0.0290 (15)	0.0049 (13)	0.0084 (14)
C12	0.074 (2)	0.097 (2)	0.0583 (19)	0.0455 (19)	0.0214 (15)	0.0207 (17)
C13	0.090 (2)	0.092 (2)	0.0431 (16)	0.0499 (19)	0.0109 (16)	0.0146 (15)
C14	0.0710 (19)	0.072 (2)	0.0510 (17)	0.0381 (17)	−0.0010 (14)	0.0069 (15)
C15	0.0579 (16)	0.0672 (19)	0.0540 (16)	0.0325 (15)	0.0094 (13)	0.0097 (14)
C16	0.0576 (16)	0.0556 (16)	0.0438 (15)	0.0283 (14)	0.0075 (13)	0.0087 (12)
C17	0.0647 (17)	0.0614 (18)	0.0443 (15)	0.0243 (16)	0.0019 (13)	0.0070 (13)
C18	0.102 (2)	0.0616 (19)	0.0517 (17)	0.0336 (18)	0.0064 (16)	0.0067 (15)
C19	0.107 (2)	0.0455 (17)	0.0530 (18)	0.0285 (18)	0.0043 (18)	0.0077 (14)
C20	0.121 (3)	0.075 (2)	0.071 (2)	0.028 (2)	0.017 (2)	0.0145 (19)
C21	0.188 (5)	0.091 (3)	0.073 (3)	0.041 (3)	0.045 (3)	0.029 (2)
C22	0.254 (7)	0.086 (3)	0.066 (3)	0.069 (4)	−0.008 (4)	0.013 (2)
C23	0.184 (5)	0.090 (3)	0.088 (3)	0.061 (3)	−0.041 (3)	0.002 (3)
C24	0.123 (3)	0.070 (2)	0.074 (2)	0.042 (2)	−0.011 (2)	0.0095 (18)

### Geometric parameters (Å, º)

**Table:**

Cl1—C4	1.735 (3)	C15—C16	1.373 (4)
Cl2—C14	1.738 (3)	C17—C18	1.520 (4)
O1—C1	1.382 (3)	C18—C19	1.506 (4)
O1—C7	1.384 (3)	C19—C20	1.375 (6)
O2—C7	1.202 (3)	C19—C24	1.377 (6)
O3—C10	1.371 (3)	C20—C21	1.388 (5)
O3—C11	1.390 (4)	C21—C22	1.343 (10)
O4—C10	1.200 (4)	C22—C23	1.361 (10)
N1—C6	1.398 (3)	C23—C24	1.376 (6)
N1—C7	1.371 (3)	C2—H2	0.9300
N1—C8	1.491 (3)	C3—H3	0.9300
N2—C8	1.430 (4)	C5—H5	0.9300
N2—C9	1.448 (4)	C8—H8A	0.9700
N2—C17	1.463 (4)	C8—H8B	0.9700
N3—C9	1.444 (3)	C9—H9A	0.9700
N3—C10	1.370 (4)	C9—H9B	0.9700
N3—C16	1.393 (3)	C12—H12	0.9300
C1—C2	1.362 (3)	C13—H13	0.9300
C1—C6	1.383 (4)	C15—H15	0.9300
C2—C3	1.381 (4)	C17—H17A	0.9700
C3—C4	1.388 (4)	C17—H17B	0.9700
C4—C5	1.387 (3)	C18—H18A	0.9700
C5—C6	1.369 (4)	C18—H18B	0.9700
C11—C12	1.360 (4)	C20—H20	0.9300
C11—C16	1.368 (4)	C21—H21	0.9300
C12—C13	1.382 (5)	C22—H22	0.9300
C13—C14	1.377 (5)	C23—H23	0.9300
C14—C15	1.386 (4)	C24—H24	0.9300
			
C1—O1—C7	107.7 (2)	C19—C20—C21	121.3 (4)
C10—O3—C11	107.1 (2)	C20—C21—C22	119.5 (5)
C6—N1—C7	109.6 (2)	C21—C22—C23	120.6 (4)
C6—N1—C8	128.6 (2)	C22—C23—C24	120.1 (6)
C7—N1—C8	121.2 (2)	C19—C24—C23	120.8 (4)
C8—N2—C9	112.5 (2)	C1—C2—H2	121.00
C8—N2—C17	114.76 (19)	C3—C2—H2	121.00
C9—N2—C17	114.5 (2)	C2—C3—H3	120.00
C9—N3—C10	123.4 (2)	C4—C3—H3	120.00
C9—N3—C16	127.5 (2)	C4—C5—H5	122.00
C10—N3—C16	108.9 (2)	C6—C5—H5	122.00
O1—C1—C2	127.6 (2)	N1—C8—H8A	108.00
O1—C1—C6	109.5 (2)	N1—C8—H8B	108.00
C2—C1—C6	122.9 (2)	N2—C8—H8A	108.00
C1—C2—C3	117.2 (2)	N2—C8—H8B	108.00
C2—C3—C4	119.5 (2)	H8A—C8—H8B	107.00
Cl1—C4—C3	118.03 (19)	N2—C9—H9A	109.00
Cl1—C4—C5	118.6 (2)	N2—C9—H9B	109.00
C3—C4—C5	123.3 (2)	N3—C9—H9A	109.00
C4—C5—C6	115.8 (2)	N3—C9—H9B	109.00
N1—C6—C1	105.7 (2)	H9A—C9—H9B	108.00
N1—C6—C5	133.2 (2)	C11—C12—H12	122.00
C1—C6—C5	121.1 (2)	C13—C12—H12	122.00
O1—C7—O2	123.0 (3)	C12—C13—H13	120.00
O1—C7—N1	107.5 (2)	C14—C13—H13	120.00
O2—C7—N1	129.6 (3)	C14—C15—H15	122.00
N1—C8—N2	115.9 (2)	C16—C15—H15	122.00
N2—C9—N3	111.3 (2)	N2—C17—H17A	109.00
O3—C10—O4	123.5 (3)	N2—C17—H17B	109.00
O3—C10—N3	108.3 (2)	C18—C17—H17A	109.00
O4—C10—N3	128.3 (3)	C18—C17—H17B	109.00
O3—C11—C12	127.0 (3)	H17A—C17—H17B	108.00
O3—C11—C16	109.4 (2)	C17—C18—H18A	109.00
C12—C11—C16	123.5 (3)	C17—C18—H18B	109.00
C11—C12—C13	115.9 (3)	C19—C18—H18A	109.00
C12—C13—C14	120.7 (3)	C19—C18—H18B	109.00
Cl2—C14—C13	118.4 (2)	H18A—C18—H18B	108.00
Cl2—C14—C15	118.6 (2)	C19—C20—H20	119.00
C13—C14—C15	123.0 (3)	C21—C20—H20	119.00
C14—C15—C16	115.1 (3)	C20—C21—H21	120.00
N3—C16—C11	106.3 (3)	C22—C21—H21	120.00
N3—C16—C15	132.1 (3)	C21—C22—H22	120.00
C11—C16—C15	121.6 (2)	C23—C22—H22	120.00
N2—C17—C18	111.8 (2)	C22—C23—H23	120.00
C17—C18—C19	112.9 (2)	C24—C23—H23	120.00
C18—C19—C20	121.2 (3)	C19—C24—H24	120.00
C18—C19—C24	121.2 (3)	C23—C24—H24	120.00
C20—C19—C24	117.7 (3)		
			
C7—O1—C1—C2	176.3 (3)	O1—C1—C2—C3	−178.1 (3)
C7—O1—C1—C6	−2.3 (3)	C2—C1—C6—N1	−178.4 (3)
C1—O1—C7—N1	3.4 (3)	C2—C1—C6—C5	−0.7 (4)
C1—O1—C7—O2	−175.8 (3)	O1—C1—C6—C5	178.0 (2)
C11—O3—C10—N3	−1.7 (3)	C1—C2—C3—C4	−0.6 (4)
C11—O3—C10—O4	179.4 (3)	C2—C3—C4—Cl1	179.3 (2)
C10—O3—C11—C16	1.8 (3)	C2—C3—C4—C5	1.3 (4)
C10—O3—C11—C12	−178.1 (3)	C3—C4—C5—C6	−1.6 (4)
C8—N1—C7—O1	−175.2 (2)	Cl1—C4—C5—C6	−179.6 (2)
C6—N1—C7—O1	−3.3 (3)	C4—C5—C6—N1	178.3 (3)
C6—N1—C8—N2	99.0 (3)	C4—C5—C6—C1	1.3 (4)
C7—N1—C8—N2	−90.7 (3)	C12—C11—C16—C15	−0.8 (5)
C7—N1—C6—C1	1.9 (3)	C12—C11—C16—N3	178.7 (3)
C8—N1—C6—C1	173.1 (3)	O3—C11—C12—C13	179.3 (3)
C7—N1—C6—C5	−175.4 (3)	C16—C11—C12—C13	−0.6 (5)
C8—N1—C6—C5	−4.3 (5)	O3—C11—C16—N3	−1.1 (3)
C6—N1—C7—O2	175.9 (3)	O3—C11—C16—C15	179.4 (2)
C8—N1—C7—O2	3.9 (5)	C11—C12—C13—C14	1.2 (5)
C17—N2—C8—N1	−70.5 (3)	C12—C13—C14—C15	−0.6 (5)
C9—N2—C17—C18	143.5 (2)	C12—C13—C14—Cl2	178.7 (3)
C8—N2—C9—N3	162.6 (2)	Cl2—C14—C15—C16	−180.0 (2)
C9—N2—C8—N1	62.8 (3)	C13—C14—C15—C16	−0.7 (4)
C8—N2—C17—C18	−84.1 (3)	C14—C15—C16—C11	1.4 (4)
C17—N2—C9—N3	−64.0 (3)	C14—C15—C16—N3	−178.0 (3)
C9—N3—C10—O3	175.6 (2)	N2—C17—C18—C19	−63.6 (3)
C16—N3—C10—O3	1.1 (3)	C17—C18—C19—C20	−74.1 (4)
C10—N3—C16—C11	0.0 (3)	C17—C18—C19—C24	106.1 (4)
C9—N3—C16—C15	5.2 (5)	C18—C19—C20—C21	−178.9 (3)
C9—N3—C10—O4	−5.6 (5)	C24—C19—C20—C21	1.0 (5)
C16—N3—C10—O4	179.9 (3)	C18—C19—C24—C23	179.0 (3)
C10—N3—C16—C15	179.5 (3)	C20—C19—C24—C23	−0.9 (5)
C10—N3—C9—N2	110.9 (3)	C19—C20—C21—C22	−1.1 (6)
C16—N3—C9—N2	−75.6 (3)	C20—C21—C22—C23	1.1 (7)
C9—N3—C16—C11	−174.2 (2)	C21—C22—C23—C24	−1.0 (7)
C6—C1—C2—C3	0.3 (4)	C22—C23—C24—C19	0.9 (6)
O1—C1—C6—N1	0.3 (3)		

### Hydrogen-bond geometry (Å, º)

**Table:**

*D*—H···*A*	*D*—H	H···*A*	*D*···*A*	*D*—H···*A*
C8—H8*A*···O4^i^	0.97	2.51	3.037 (4)	114
C9—H9*A*···O4	0.97	2.56	2.921 (4)	102
C17—H17*A*···N3	0.97	2.54	2.944 (3)	105

Symmetry code: (i) *x*−1, *y*, *z*.
